# Simultaneous standard total joint prosthesis reconstruction with SSRO and Le Fort I osteotomy in the treatment of unilateral temporomandibular joint ankylosis with jaw deformity: a case cohort study

**DOI:** 10.1007/s00784-024-05543-3

**Published:** 2024-02-21

**Authors:** Dong Huang, Luxiang Zou, Chuan Lu, Jieyun Zhao, Dongmei He, Chi Yang

**Affiliations:** 1 Department of Oral Surgery, Shanghai Ninth People’s Hospital, Shanghai Jiao Tong University School of Medicine, College of Stomatology, Shanghai Jiao Tong University, Shanghai, 200011 China; 2 National Center for Stomatology, National Clinical Research Center for Oral Diseases, Shanghai Key Laboratory of Stomatology, Shanghai Research Institute of Stomatology, Shanghai, 200011 China

**Keywords:** Temporomandibular joint ankylosis, Jaw deformity, Total joint prosthesis, Sagittal split ramus osteotomy, Le Fort I osteotomy, Computer-assisted surgery

## Abstract

**Objective:**

Unilateral temporomandibular joint ankylosis with jaw deformity (UTMJAJD) may require simultaneous total joint prosthesis (TJP) reconstruction, sagittal split ramus (SSRO), and Le Fort I osteotomies. The purpose of this study was to evaluate outcomes in patients treated with these procedures.

**Methods:**

Patients diagnosed UTMJAJD between 2016 and 2018 were selected for the study. Mandible-first procedure was performed after ankylosis release with TJP on the ankylosed side and SSRO on the contralateral side. Le Fort I osteotomy with and without genioplasty was lastly performed. Maximal incisor opening (MIO), facial symmetry, and jaw and condyle stability were compared before, after operation, and during follow-ups.

**Results:**

Seven patients were included in the study. Their average chin deviation was 9.5 ± 4.2 mm, and maxillary cant was 5.1 ± 3.0°. After operation, jaw deformity significantly improved, with chin deviation corrected 7.6 ± 4.1 mm (*p* = 0.015) and advanced 5.9 ± 2.5 mm (*p* = 0.006). After an average follow-up of 26.6 ± 17.1 months, MIO significantly increased from 11.4 ± 9.3 to 35.7 ± 2.6 mm (*p* = 0.000). The occlusion was stable with no significant positional or rotational changes of the jaw (*p* > 0.05). There was no obvious condylar resorption during follow-ups.

**Conclusion:**

Simultaneous TJP reconstruction, SSRO, and Le Fort I osteotomy are reliable and effective methods for the treatment of UTMJAJD.

## Introduction

Temporomandibular joint ankylosis (TMJA) is a severe joint disease that is mainly caused by trauma or infection. It can affect masticatory function, digestion, speech, as well as oral hygiene [1–5]. When it occurs in childhood, dentofacial deformities and obstructive sleep apnea syndrome (OSAS) may develop [6–11]. Surgery is the only treatment for TMJA. According to our previous classification [3], patients who had no joint structures or insufficient functional residual condyles require joint reconstruction. Total joint prosthesis (TJP) has gradually replaced autogenous bone graft for TMJ reconstruction with the advantages of no resorption and low relapse rates [12, 13].

For patients with jaw deformities, joint reconstruction is combined with orthognathic surgery simultaneously or secondarily [5]. We have reported using standard TJP with Le Fort I osteotomy to correct bilateral TMJA with severe mandibular deficiency [12–14]. However, for patients with unilateral TMJA with jaw deformity (UTMJAJD), the method of joint reconstruction and stability of the contralateral healthy joint with orthognathic surgery has not been reported.

The aim of this study was to introduce a surgical method for UTMJAJD under the guide of computer-assisted surgery (CAS) and to evaluate its stability by three-dimensional measurement.

## Materials and methods

### Patient selection

This is a retrospective study approved by the Ethics Committee of Ninth People’s Hospital Affiliated to Shanghai Jiao Tong University School of Medicine (SH9H-2018-T37-1). Patients diagnosed with UTMJAJD by CT from November 2016 to November 2018 were recruited. The inclusion criteria were as follows: (1) age over 18 years; (2) UTMJA without residual functional condyle; (3) TMJ reconstruction using prostheses; and (4) simultaneous orthognathic surgery including SSRO on the contralateral side and Le Fort I osteotomy ± genioplasty to correct jaw deformity. The exclusion criteria were (1) incomplete clinical and CT data before operation, within 1 week after operation or during follow-ups; (2) previous contralateral joint surgery; and (3) follow-up periods less than 6 months.

### Preoperative measurements and virtual surgical planning

Before surgery (T0), all patients underwent CT scans with a slice thickness of 0.625 mm (GE Healthcare, Chicago, IL). The scanning data was saved in Digital Imaging and Communications in Medicine (DICOM) format for further use. CAS procedure including deformity measurement and surgery design was performed under the supervision of both surgeons and orthodontists. All patients were planned to receive a surgery-first procedure, without post-operative orthodontic treatment.

**Fig. 1:**
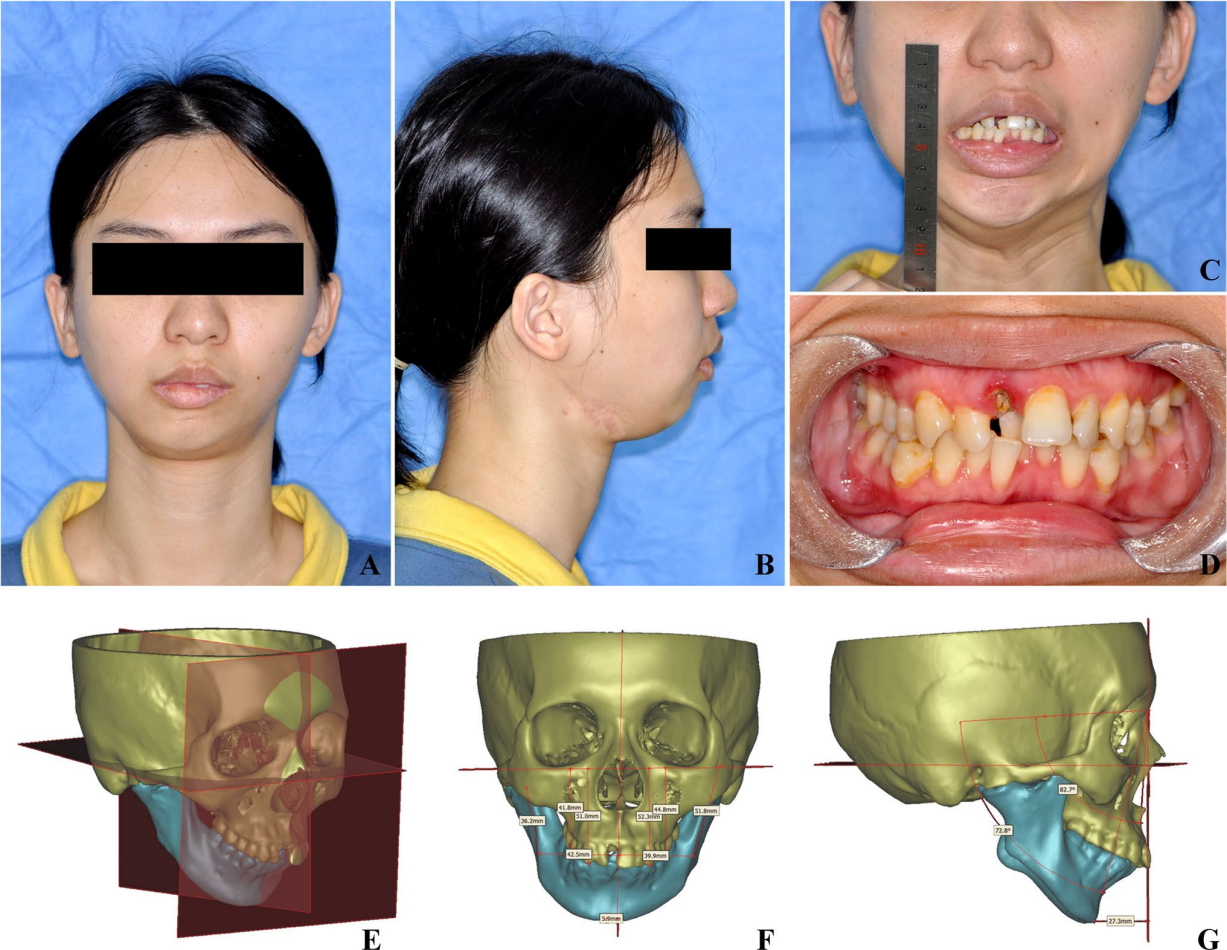
Pre-operative evaluation. **A**–**D** Clinical evaluation including facial appearance, MIO, and occlusion. **E**–**G** Coordinate system and cephalometry (patient 5)

Three-dimensional reconstruction and cephalometry were performed using Proplan CMF 1.4 software (Materialize, Leuven, Belgium). The definitions of cephalometry landmarks are listed in Table 1, and pre-operative measurements were recorded as T0. A coordinate system was established, with the X plane (FH plane or horizontal plane) passing through OrL, OrR, and the middle point of bilateral porion points (PoMid); the Y plane (sagittal plane) passing through N, S, and Ba; and the Z plane (coronal plane) passing through N and perpendicular to the X and Y planes. Preoperative procedures included (Fig. 1):

**Table 1:** Measurement definition

Values	Definition
**Coordinate system**	
FH plane (X plane)	A plane passes bilateral orbital point (OrL, OrR) and the middle point of bilateral porion points (PoMid)
Middle Sagittal plane (Y plane)	A plane passes nasal point (N), anterior margin of foramen occipitale magnum (Ba), and fossa hypophysialis (S)
Coronal plane (Z plane)	A plane passes nasal point (N) and perpendicular to X plane and Y plane
**Maxillary stability**	
SNA (°)	Angle of sella-nasal-subspinale (A point)
Central incisor distance (CI, mm)	Distance between central incisor (CI) point and FH plane
Cant 3 (mm)	Difference between distances of upper canine cusp points (U3L/R) and FH plane
Cant 6 (mm)	Difference between distances of upper first molar medial-buccal cusp points (U6L/R) and FH plane
Maxillary cant angle (°)	Projection of the angle between maxilla occlusal plane (defined by CI, U6L, and U6R) and FH plane on Z plane
MP-FH (°)	Angle between maxilla occlusal plane (defined by CI, U6L, and U6R) and FH plane
Maxilla pitch (°)	Angle between maxilla pitch plane (defined by A, CI and perpendicular to mid-sagittal plane) and X plane
Maxilla roll (°)	Angle between maxilla roll plane (defined by U6L, U6R and perpendicular to coronal plane) and Y plane
Maxilla yaw (°)	Angle between maxilla yaw plane (defined by U6L, U6R and perpendicular to FH plane) and Z plane
**Mandibular stability**	
SNB (°)	Angle of sella-nasal-supramental (B point)
Chin deviation (mm)	Distance between gnathion (Gn) and mid-sagittal plane
Chin retrusion (mm)	Distance between gnathion (Gn) and Z plane
**Joint stability**	
Ramus height (RH, mm)	Distance between condyle point (CoL/R) and gonion point (Go)
Ramus height correct (RH′, mm)	Distance between condyle point and anterior notch of mandibular angle. Used to correct the condyle height value on the alloplastic TJP side
Condyle pitch (°)	Angle between condyle pitch plane (defined by anterior condyle-CoA, posterior condyle-CoP, and perpendicular to Y plane) and FH plane
Condyle roll (°)	Angle between condyle roll plane (defined by lateral condyle-CoLat, medial condyle-CoMed and perpendicular to Z plane) and Y plane
Condyle yaw (°)	Angle between condyle yaw plane (defined by CoLat, CoMed and perpendicular to X plane) and Z plane
Co-X (mm)	Distance between contralateral Co point and the FH plane
Co-Y (mm)	Distance between contralateral Co point and the Y plane
Co-Z (mm)	Distance between contralateral Co point and Z plane
CoLat-Y (mm)	Distance between contralateral CoLat point and the Y plane
CoMed-Y (mm)	Distance between contralateral CoMed points and the Y plane
CoA-Z (mm)	Distance between contralateral CoA points and the Z plane
CoP-Z (mm)	Distance between contralateral CoP points and the Z plane

### Jaw symmetry measurement

Maxillary symmetry was assessed based on maxillary cant, which is the difference between the distances of the upper canine cusp points or upper first molar medial-buccal cusp points and the FH plane. The angle between the maxillary occlusal plane and FH plane was also measured.

Mandible symmetry was assessed based on several parameters, including chin deviation (distance between Gn and Y Plane), chin retrusion (distance between Gn and Z Plane), and ramus height (RH) on both sides.

These parameters are used to evaluate the symmetry of the jaw, and to help evaluate the outcome of the surgery.

In addition, the ankylosed bony mass was marked and measured to determine the optimal region and depth for the osteotomy. This approach ensured that important anatomical structures were protected from potential damage during the surgery.

### Virtual surgery design and splint manufacture

**Fig. 2:**
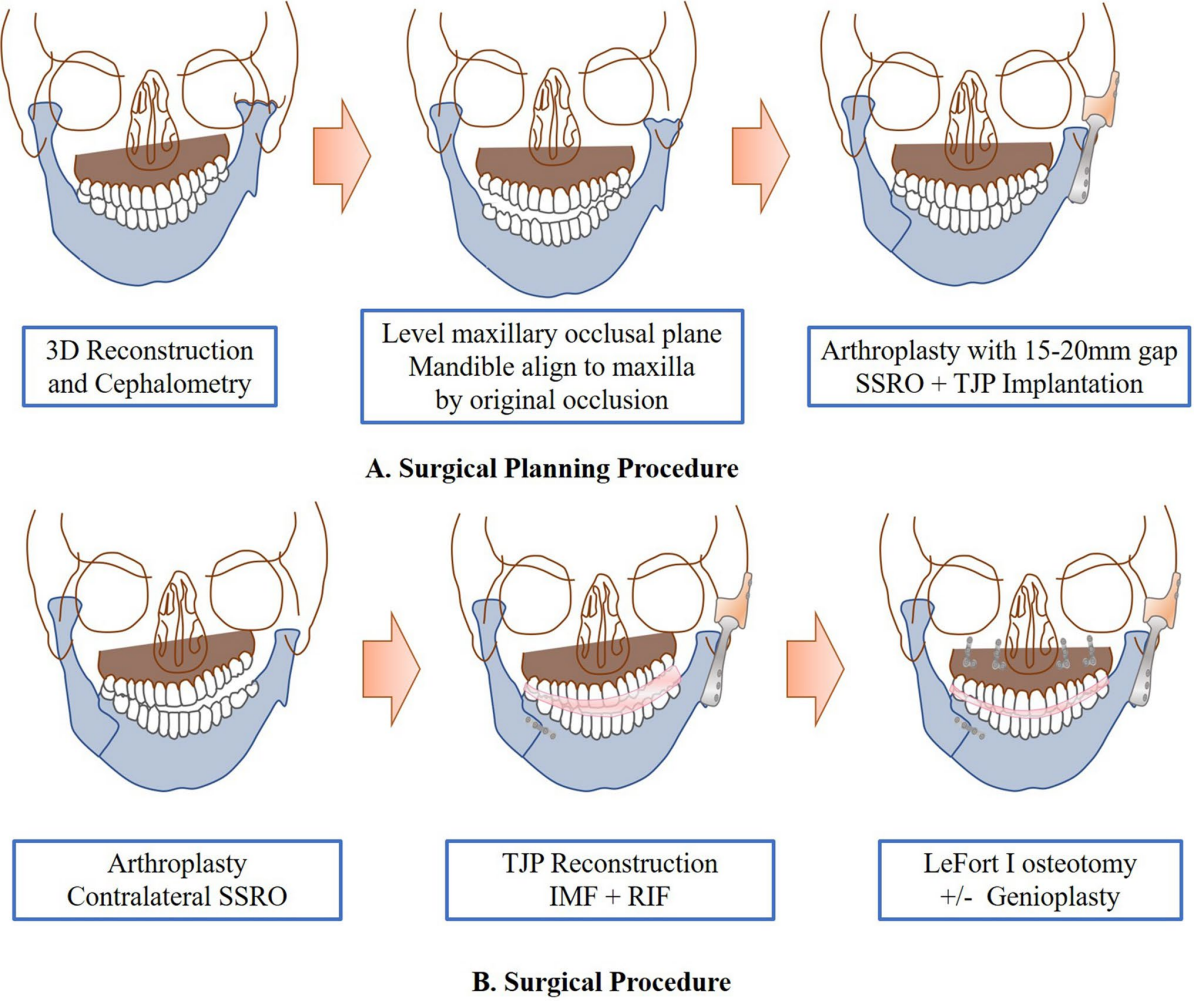
Virtual and actual surgical procedures. **A** Surgical planning procedure. **B** Surgical procedure

The virtual surgery performed consisted of several steps (Fig. 2A): (1) Le Fort I osteotomy to level the maxillary occlusal plane with 1–2-mm overcorrection at the first molar; (2) resection of the ankylosed bony mass with a minimum gap of 15–20 mm between the mandibular ramus stump and the articular fossa; (3) TJP implantation and contralateral SSRO to level the mandible and maintain the original occlusion; and (4) genioplasty was selected to further correct chin deviation and retrusion if necessary.

An intermediate occlusal splint of mandible first was designed and manufactured. Additionally, guiding templates were fabricated for the resection of the ankylosed bony mass, and the accuracy of the TJP implantation.

### Surgical procedure

**Fig. 3:**
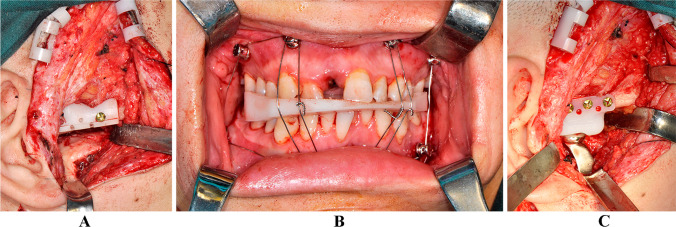
Operation procedures (patient 5). **A** Application of osteotomy guide. **B** IMF using mandible first intermediate occlusal splint. **C** Implantation of TJP

Modified preauricular [15] and submandibular incisions were performed to access the bony fusion and ramus. An osteotomy guide was used to aid the bony fusion resection and the coronoid process removal (Fig. 3A). To prevent re-ankylosis and enable the implantation of the fossa prosthesis, a gap of at least 15–20 mm between the mandibular ramus stump and fossa was necessary. After sealing the joint region and disinfecting the oral cavity, the contralateral coronoid process was removed if mouth opening was less than 35 mm. Subsequently, SSRO was conducted to correct the mandibular asymmetry. The bony fragments were fixed after intermaxillary fixation (IMF) with the intermediate occlusal splint (Fig. 3B). The oral cavity was then sealed, and the facial area was disinfected and draped. Surgical gowns and gloves were changed. After the implantation of the TJP, the gap around the prosthesis was filled with fat obtained from the abdomen to prevent heterotopic ossification (HO), thereby reducing the rate of re-ankylosis (Fig. 3C) [16, 17]. The surgical incision was closed in layers with a drain placed inside the wound. Finally, Le Fort I osteotomy was performed to restore the final occlusion. Genioplasty was selected for correction of chin retrusion and deviation if necessary. The actual surgical procedures are shown in Fig. 2B.

### Postoperative and follow-up evaluations

All patients underwent CT scans within 1 week after the operation (T1) and during follow-up visits. The final follow-up visit was defined as T2. Three-dimensional reconstruction and cephalometry were performed in both T1 and T2, using the same coordinate system and measurements as in T0. The evaluations were conducted by two different experienced doctors within a 2-week time interval. The evaluations were performed as follows:

### Clinical evaluation

At the last follow-up visit, the facial symmetry, MIO, and symptoms of contralateral joints of all patients were recorded (Fig. 6). Additionally, the recurrence of ankylosis was evaluated and recorded via clinical examinations and CT scans.

### Jaw symmetry evaluation

The symmetry of the maxilla and mandible was measured and evaluated according to preoperative methods, including maxillary cant, chin deviation, and retrusion, and the ramus height on both sides using CT scans taken post-operation and at the last follow-up visit.

### Jaw stability evaluation

Jaw stability was evaluated in terms of position and rotation between the postoperative and follow-up measurements. Distances and angles of cephalometry demonstrated the position of bones, while the angles between specific planes and coordinate reference planes demonstrated the rotation of bones.

Maxillary position was evaluated by SNA, CI distance, and maxillary cant on canines and first molars. Maxillary rotation was defined and evaluated according to angles between the occlusal plane and FH plane (MP-FH), as well as maxillary roll, pitch, and yaw angles (Table 1).

Mandibular position was evaluated by SNB, chin deviation, and retrusion. Ramus height was also measured (Co-Go), and measurements were corrected for affected sides (distance between the condyle point and anterior notch of mandibular angle) to avoid error caused by bony changes in the tuberositas masseterica region and posterior border of the ramus.

### Contralateral condyle stability evaluation

**Fig. 4:**
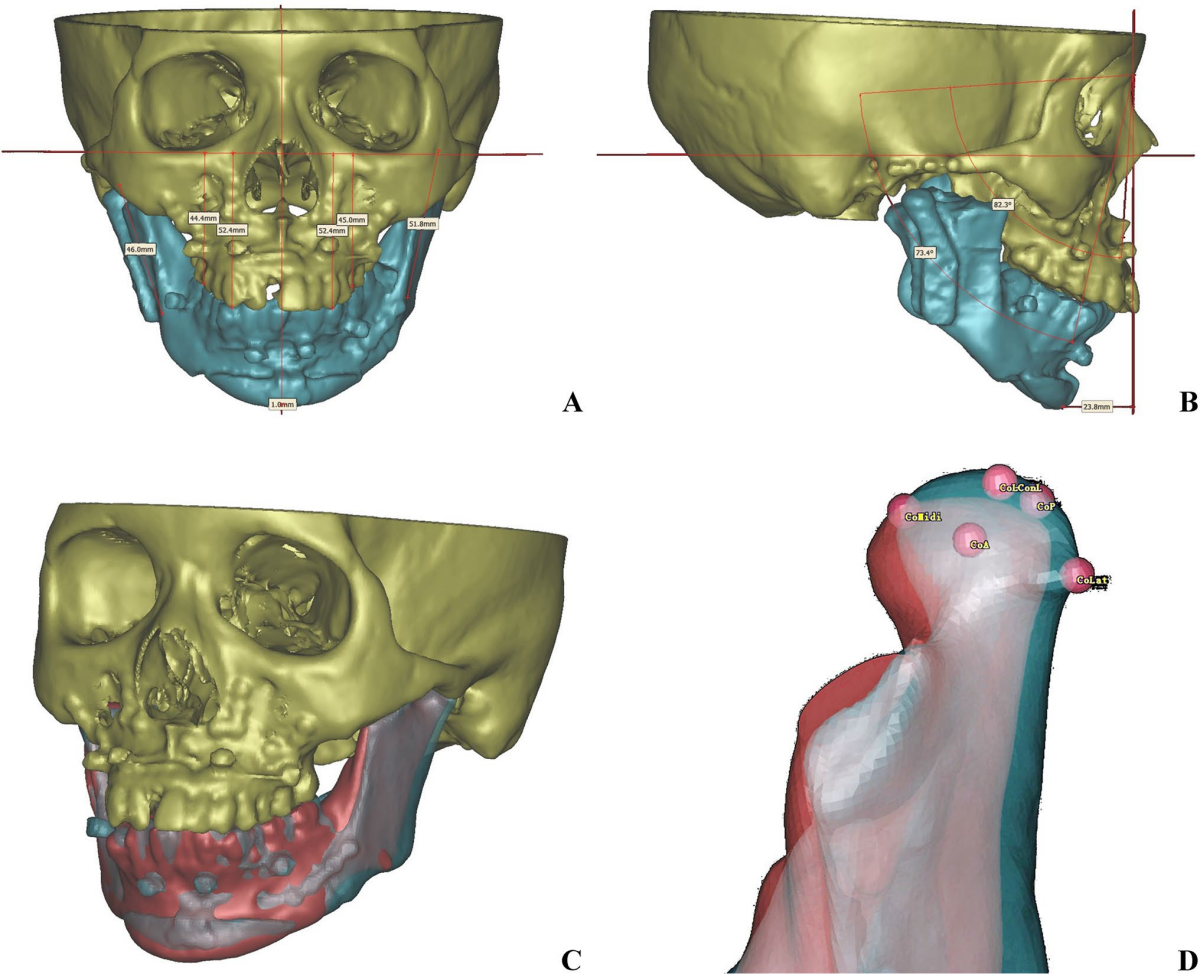
Post-operation (T1) CT image and comparison of positions between T1 (blue) and T2 (red) (patient 5). **A**, **B** Post-operation 3D reconstruction and cephalometry. **C**, **D** Comparison of mandibular and condylar position between T1 and T2

The contralateral healthy condyle stability was evaluated by rotation, position, and bone remodeling. Condylar rotation was evaluated by pitch, roll, and yaw angles (as defined in Table 1 and Fig. 4); condyle position was evaluated by distances between landmarks (condylar top, medial, lateral, anterior, and posterior surface points) and X, Y, and Z planes. The remodeling of the surface bone was compared by superimposition comparison using surface-best-fit registration according to non-surgical areas of the mandible between T1 and T2. The comparison threshold was set as 2 mm [18].

### Statistical analysis

Jaw position and condylar surface bony changes between T0, T1, and T2 were compared, respectively, using a paired *t* test with SPSS 19.0 software package (IBM, USA). A *p* value of less than 0.05 was considered statistically significant.

## Results

Seven patients were included in the study. They were all females with an average age of 28.4 ± 15.5 years (ranging from 19 to 53 years). Their mean duration of ankylosis was 21.0 ± 11.4 years (ranging from 11 to 45 years). One patient had ankylosis due to infection, while the other six were caused by trauma. Five patients were on the right sides and two on the left. Their preoperative MIO was 11.4 ± 9.3 mm (ranging from 0 to 25 mm, shown in Table 2).

**Table 2:** Basic information of the patients

No	Gender	Age	Duration (years)	Cause	Side	Pre-op MIO (mm)	Operation	FU (months)	FU MIO (mm)
1	Female	30	22	Infection	Right	0	TJP + SSRO + LFI	62	32
2	Female	53	45	Trauma	Left	25	TJP + SSRO + LFI + GP	7	35
3	Female	19	13	Trauma	Right	15	TJP + SSRO + LFI	27	40
4	Female	23	20	Trauma	Right	5	TJP + SSRO + LFI + GP	23	35
5	Female	25	21	Trauma	Right	2	TJP + SSRO + LFI + GP	26	34
6	Female	28	15	Trauma	Right	15	TJP + SSRO + LFI	24	36
7	Female	21	11	Trauma	Left	18	TJP + SSRO + LFI	17	38
**Average**	**-**	28.4 ± 15.5	21.0 ± 11.4	**-**	**-**	11.4 ± 9.3*	**-**	26.6 ± 17.1	35.7 ± 2.6*

*Pre-op* pre-operation, *MIO* maximal incisal opening, *FU* follow-up, *TJP* total joint prosthesis, *SSRO* sagittal split ramus osteotomy, *LFI* Le Fort I osteotomy, *GP* genioplasty

^*^p = 0.000 < 0.05

Three-dimensional measurements showed that before operation, the mean chin deviation was 9.5 ± 4.2 mm (ranging from 6.0 to 15.3 mm), and retrusion was 29.1 ± 9.1 mm (ranging from 17.9 to 42.6 mm). The maxillary cant was 2.4 ± 1.2 mm on the canines (ranging from 1.3 to 4.5 mm) and 4.5 ± 2.9 mm on the first molars (ranging from 1.8 to 9.3 mm), with a cant angle of 5.1 ± 3.0° (ranging from 2.1 to 9.7°) (Table 3).

**Table 3:** Statistical comparison between T0, T1, and T2

	T0	T1	T2	*p*_T0-T1_ value	*p*_T1-T2_ value
**Maxillary stability**					
SNA (°)	82.5 ± 4.3	80.9 ± 4.9	80.2 ± 5.4	0.166	0.068
CI (mm)	54.0 ± 2.6	52.7 ± 1.6	53.2 ± 1.5	0.214	0.831
Cant 3 (mm)	2.4 ± 1.2	0.7 ± 0.5	0.6 ± 0.4	0.019*	0.227
Cant 6 (mm)	4.5 ± 2.9	1.0 ± 0.6	1.7 ± 0.9	0.030*	0.071
Maxilla cant angle (°)	5.1 ± 3.0	1.1 ± 0.6	2.0 ± 0.9	0.022*	0.051
MP-FH (°)	18.7 ± 1.6	12.1 ± 3.3	13.0 ± 2.2	0.016*	0.561
Maxilla-pitch (°)	-	80.6 ± 5.0	79.1 ± 5.1	-	0.188
Maxilla-roll (°)	-	88.8 ± 0.6	88.1 ± 1.2	-	0.057
Maxilla-yaw (°)	-	2.8 ± 1.8	3.5 ± 3.0	-	0.385
**Mandiblular stability**					
SNB (°)	70.7 ± 6.3	72.4 ± 4.2	73.0 ± 4.8	0.214	0.289
Chin deviation (mm)	9.5 ± 4.2	1.9 ± 1.4	1.7 ± 1.3	0.015*	0.442
Chin retrusion (mm)	29.1 ± 9.1	23.2 ± 7.3	22.4 ± 7.8	0.006*	0.517
**Joint stability**					
RH-affected side (mm)	42.4 ± 14.8	51.3 ± 4.7	-	0.174	-
RH non-affected side (mm)	57.4 ± 4.9	57.2 ± 4.5	57.3 ± 5.9	0.480	0.884
RH′ affected side (mm)	-	54.8 ± 2.9	57.2 ± 6.2	-	0.804
Co-X (mm, non-affected side)	-	1.6 ± 1.3	2.1 ± 2.2	-	0.332
Co-Y (mm, non-affected side)	-	52.4 ± 3.5	42.5 ± 19.8	-	0.265
Co-Z (mm, non-affected side)	-	69.6 ± 4.5	67.9 ± 5.5	-	0.275
CoLat-Y (mm, non-affected side)	-	58.6 ± 4.3	47.7 ± 22.3	-	0.266
CoMed-Y (mm, non-affected side)	-	42.3 ± 1.0	33.7 ± 15.3	-	0.276
CoA-Z (mm, non-affected side)	-	64.4 ± 4.9	62.1 ± 5.7	-	0.131
CoP-Z (mm, non-affected side)	-	74.4 ± 3.5	72.6 ± 4.9	-	0.271
Condyle roll (°, non-affected side)	-	80.3 ± 5.7	81.8 ± 6.2	-	0.563
Condyle yaw (°, non-affected side)	-	12.7 ± 3.3	8.1 ± 3.6	-	0.122
Condyle pitch (°, non-affected side)	-	6.0 ± 3.5	5.6 ± 3.1	-	0.835

^*^p < 0.05

After operation, the ramus height on the affected side increased by 9.2 ± 11.7 mm, the chin deviation was corrected 7.6 ± 4.1 mm (*p* = 0.015), and advanced 5.9 ± 2.5 mm (*p* = 0.006) significantly. Maxillary cant was corrected 1.7 ± 1.0 mm at the canines (*p* = 0.02) and 3.5 ± 2.4 mm at the first molars (*p* = 0.03). The maxillary cant angle was corrected 4.0 ± 2.4° (*p* = 0.02) (Table 3).

**Fig. 5:**
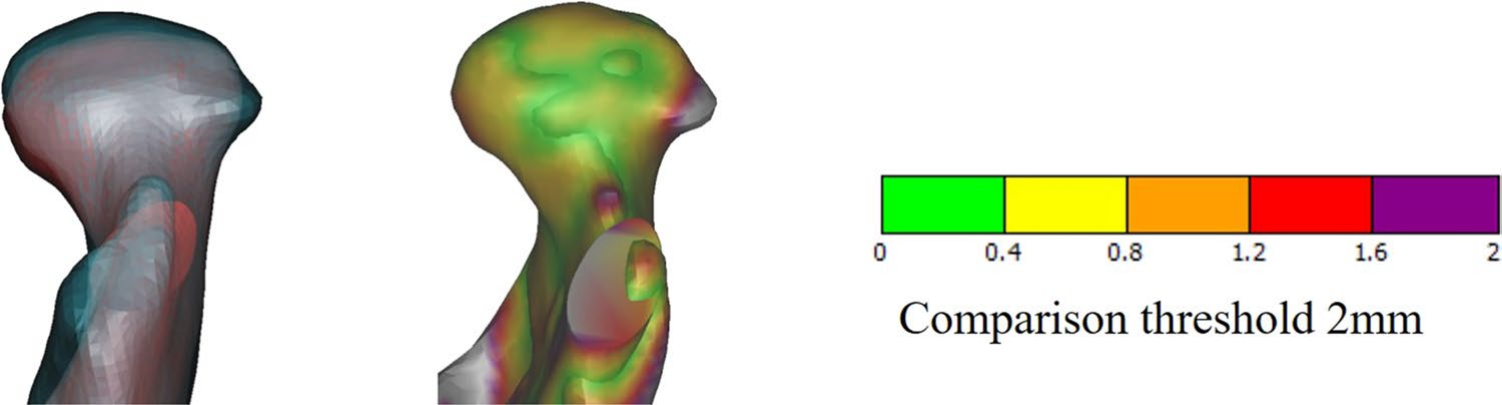
Superimposition comparison of healthy condyle between T1 (blue) and T2 (red) with a threshold of 2 mm (patient 5). No obvious bony resorption was found

After an average of 29.0 ± 20.1 months follow-up (ranging from 7 to 62 months), no recurrence of ankylosis occurred. MIO was significantly improved to 35.7 ± 2.6 mm (ranging from 32 to 40 mm, *p* = 0.000 < 0.05). No patient reported joint pain or occlusion changes. There were no significant position changes of both the maxilla and the mandible (*p* > 0.05, Table 3). The contralateral healthy condyle had 4.6 ± 5.2° of yaw rotation, but the difference was not statistically significant (*p* = 0.122 > 0.05, Table 3). There was no obvious condylar surface bone resorption at the last follow-up (Fig. 5).

## Discussion

There are two types of TMJA according to our previous classification for treatment method selection: with and without residual condyle [3]. For the residual condyle which is larger than one-half of the healthy side, lateral gap arthroplasty (LAP) is selected, while for the small or no residual condyle, autogenous bone graft or TJP is recommended for joint reconstruction. Compared with commonly used autogenous bone graft for joint reconstruction such as costochondral graft (CCG), coronoid process graft (CPG), and distraction osteogenesis (DO), which had excellent histocompatibility and reasonable cost [5], TJP is more and more used because of stability and predictability, especially for patients who need large mandibular advancement to correct jaw deformity [19]. We have reported using standard TJP and Le Fort I osteotomy simultaneously for bilateral TMJA with severe mandibular deficiency [14]. Their mean chin advancement was 10.19 mm with significantly improved SNB and ramus heights. After a mean follow-up period of 22 months, the results were stable.

However, for UTMJAJD patients, the outcome of joint reconstruction with orthognathic surgery to the contralateral healthy condyle is unknown. It was reported that about 30% of patients who received TJP treatment experienced symptoms on the contralateral temporomandibular joint [20]. In patients with jaw asymmetry, the articular disc experiences an overload of force, which could lead to disc displacement [21]. After BSSO, the forces on the condyles may become balanced due to the correction of facial asymmetry. But in UTMJAJD patients, there are currently no reports on the influence of TJP implantation with simultaneous SSRO and Le Fort I osteotomy on the contralateral healthy condyle. Results of our study showed an average mandibular ramus elongation of 9.2 ± 11.7 mm, with chin deviation correction of 7.6 ± 4.1 mm, and chin advancement of 5.9 ± 2.5 mm. However, these significant changes did not lead to condylar resorption on the contralateral sides, and no positional or rotational changes were found in follow-ups. The possible reason for this may be that SSRO reduces the force accumulation on the healthy side condyle.

For patients with TMJA and mild-to-moderate deformities, a one-stage surgical procedure can be a suitable option, with the advantages of immediate satisfaction and low cost. However, in those cases with severe dentofacial deformities, staged treatment is necessary for optimal results [5]. In this study, unilateral TJP reconstruction with simultaneous SSRO and Le Fort I osteotomy had a stable outcome in UTMJAJD patients, which can be attributed to the application of CAS technique. First, 3D cephalometry was performed to thoroughly evaluate facial asymmetry, which led to a more accurate treatment plan. There were differences in surgical planning and operation procedures for these patients. In virtual surgery, the level of the occlusal plane was the first step. Then, the ankylotic bone release, TJP implantation, and SSRO were designed. However, during the actual operation, the ankylosis release was the first step, in order to remove the bony mass and to gain mobility of the mandible, so a mandible-first procedure was necessary. After removing the ankylosed bony mass, SSRO was performed on the contralateral side. The intermediate occlusal splint determined by initial maxillary position and final mandibular position was then inserted prior to SSRO fixation and implantation of TJP. Additionally, the installation of the prosthesis and Le Fort I osteotomy were guided by custom-made templates, which improved the surgical accuracy.

**Fig. 6:**
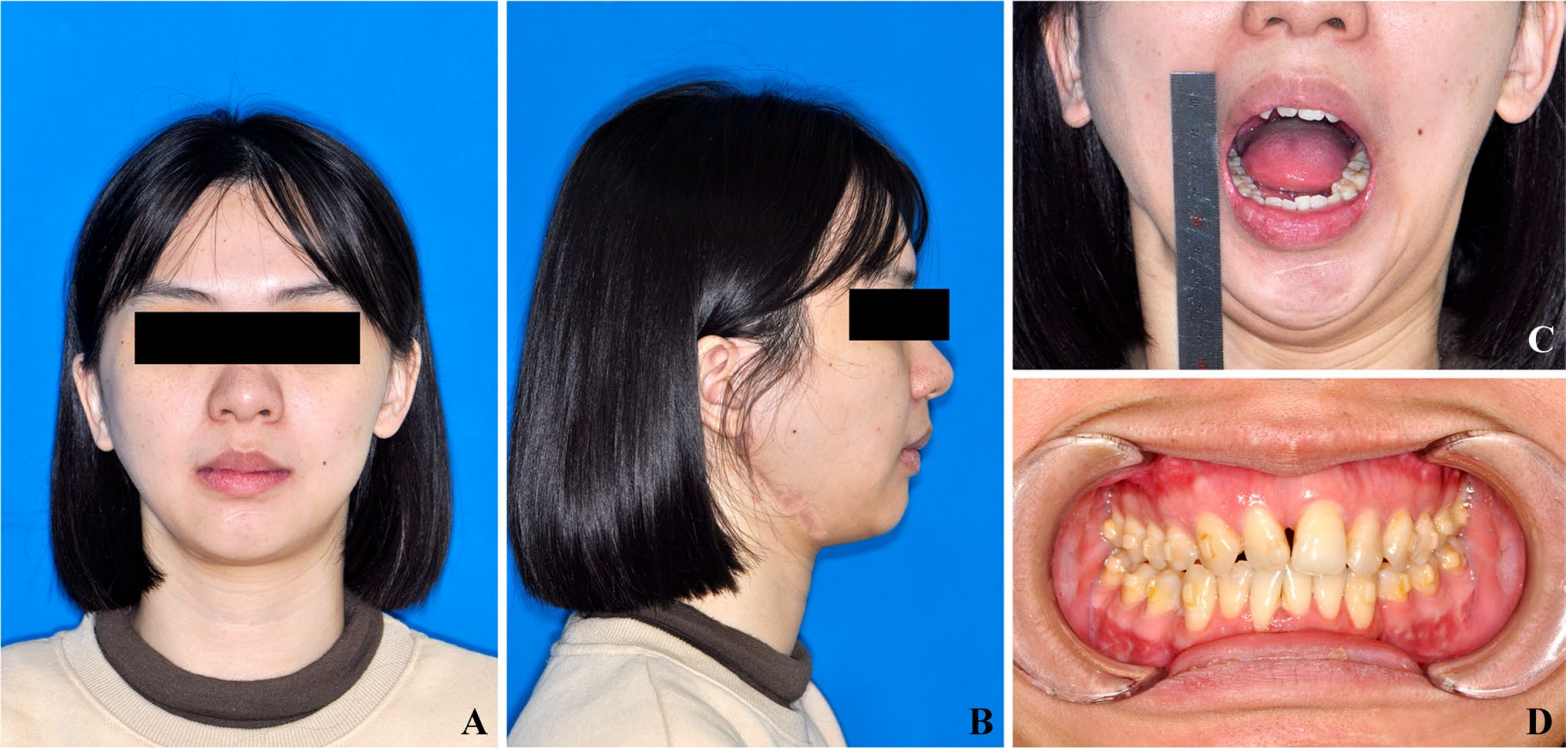
The last follow-up result of patient 5. **A** Frontal view. **B** Lateral view. **C** MIO. **D** Occlusion

We used the method as we previously reported to assess jaw stability after simultaneous TMJ and orthognathic surgery, which includes jaw position, rotation, contralateral healthy condyle position, and surface bone remodeling [18]. The results showed that at the last follow-up, jaw and condylar position were stable, and no obvious bone resorption happened on the condylar bone surface (Fig. 6). There were no patients that developed recurrent ankylosis. The occlusion was stable. However, being a preliminary study, the sample size and follow-up period were limited. It is well known that TMJ prostheses work over a few years but often not for 10 and more years due to several factors [22]. Infection is the main cause of prosthesis failure within the first 6 months after surgery. HO formation, wear or fracture of the prosthesis, foreign body reaction, and allergy to the prosthesis are the common reasons for prosthesis revision [23]. The patients should be informed and understand the necessary of revision surgery to extend the implantation periods [22]. In this study, the results provide encouragement for the future extension of this simultaneous TJR and orthognathic treatment method to a larger number of patients.

In conclusion, the combination of TJP, SSRO, and Le Fort I osteotomy for the simultaneous treatment of UTMJAJD is reliable and effective.

## Data Availability

The datasets used and/or analyzed during the current study are available from the corresponding author on reasonable request.
